# Supplementary material for: Antibiotic Susceptibility, Virulome, and Clinical Outcomes in European Infants with Bloodstream Infections Caused by Enterobacterales
Source: Antibiotics (Basel). 2021 Jun 11;10(6):706. doi: 10.3390/antibiotics10060706 (PMC8230887; doi:10.3390/antibiotics10060706)
Supplement: Supplementary file 1 [file antibiotics-10-00706-s001.zip › antibiotics-1238301-supplementary.pdf]

Supplementary Table 1: Isolate-specific accession numbers

Isolate code	Species	Study	Country	Date of sample	Accession number
econeo_1	*Escherichia coli*	CLAHRC	UK	06/06/2015	ERS6404384
econeo_2	*Escherichia coli*	CLAHRC	UK	15/03/2015	ERS6404385
econeo_3	*Escherichia coli*	CLAHRC	UK	12/03/2015	ERS6404326
koxneo_4	*Klebsiella oxytoca*	CLAHRC	UK	22/02/2015	ERS6404327
econeo_5	*Escherichia coli*	CLAHRC	UK	18/03/2015	ERS6404328
slineo_6	*Serratia liquefaciens*	CLAHRC	UK	25/02/2015	ERS6404334
econeo_7	*Escherichia coli*	CLAHRC	UK	13/10/2014	ERS6404337
econeo_8	*Escherichia coli*	CLAHRC	UK	28/10/2014	ERS6404336
econeo_9	*Escherichia coli*	CLAHRC	UK	02/02/2015	ERS6404333
eclneo_10	*Enterobacter cloacae*	NEOMERO1	Estonia	04/09/2012	ERS6404339
eclneo_11	*Enterobacter cloacae*	NEOMERO1	Estonia	05/09/2012	ERS6404329
econeo_12	*Escherichia coli*	NEOMERO1	Estonia	26/09/2012	ERS6404331
easneo_13	*Enterobacter asburiae*	NEOMERO1	Estonia	04/10/2012	ERS6404330
koxneo_14	*Klebsiella oxytoca*	NEOMERO1	Estonia	10/11/2012	ERS6404332
koxneo_15	*Klebsiella oxytoca*	NEOMERO1	Estonia	21/05/2013	ERS6404338
eclneo_16	*Enterobacter cloacae*	NEOMERO1	Estonia	31/07/2013	ERS6404335
koxneo_17	*Klebsiella oxytoca*	NEOMERO1	Estonia	01/09/2013	ERS6404341
eclneo_18	*Enterobacter cloacae*	NEOMERO1	Estonia	27/09/2013	ERS6404340
koxneo_19	*Klebsiella oxytoca*	NEOMERO1	Estonia	30/09/2013	ERS6404345
econeo_20	*Escherichia coli*	NEOMERO1	Estonia	20/01/2014	ERS6404342
easneo_21	*Enterobacter asburiae*	NEOMERO1	Estonia	11/02/2014	ERS6404346
koxneo_22	*Klebsiella oxytoca*	NEOMERO1	Estonia	12/02/2014	ERS6404343
eclneo_23	*Enterobacter cloacae*	NEOMERO1	Estonia	27/04/2014	ERS6404344
eclneo_24	*Enterobacter cloacae*	NEOMERO1	Estonia	02/04/2013	ERS6404359
eclneo_25	*Enterobacter cloacae*	NEOMERO1	Estonia	22/07/2013	ERS6404360
eclneo_26	*Enterobacter cloacae*	NEOMERO1	Estonia	31/07/2013	ERS6404357
eclneo_27	*Enterobacter cloacae*	NEOMERO1	Estonia	23/08/2013	ERS6404351
pmineo_28	*Proteus mirabilis*	NEOMERO1	Estonia	26/03/2014	ERS6404356
kpnneo_29	*Klebsiella pneumoniae*	NEOMERO1	Estonia	19/06/2014	ERS6404350
eclneo_30	*Enterobacter cloacae*	NEOMERO1	Estonia	01/11/2014	ERS6404349

easneo_31	*Enterobacter asburiae*	NEOMERO1	Spain	20/04/2013	ERS6404352
kpnneo_32	*Klebsiella pneumoniae*	NEOMERO1	Greece	25/09/2013	ERS6404348
kpnneo_33	*Klebsiella pneumoniae*	NEOMERO1	Greece	31/01/2014	ERS6404362
kpnneo_34	*Klebsiella pneumoniae*	NEOMERO1	Greece	02/02/2014	ERS6404358
kpnneo_35	*Klebsiella pneumoniae*	NEOMERO1	Greece	29/07/2014	ERS6404363
eclneo_36	*Enterobacter cloacae*	NEOMERO1	Greece	22/08/2014	ERS6404365
eaeneo_37	*Enterobacter aerogenes*	NEOMERO1	Greece	02/09/2014	ERS6404364
kpnneo_38	*Klebsiella pneumoniae*	NEOMERO1	Greece	10/09/2012	ERS6404361
econeo_39	*Escherichia coli*	NEOMERO1	Italy	15/07/2013	ERS6404374
smaneo_40	*Serratia marcescens*	NEOMERO1	Italy	24/07/2013	ERS6404372
econeo_41	*Escherichia coli*	NEOMERO1	Italy	26/09/2013	ERS6404379
kpnneo_42	*Klebsiella pneumoniae*	NEOMERO1	Italy	10/10/2013	ERS6404380
kpnneo_43	*Klebsiella pneumoniae*	NEOMERO1	Italy	08/09/2013	ERS6404376
eaeneo_44	*Enterobacter aerogenes*	NEOMERO1	Italy	01/10/2013	ERS6404367
econeo_45	*Escherichia coli*	NEOMERO1	Italy	15/02/2014	ERS6404353
econeo_46	*Escherichia coli*	NEOMERO1	Lithuania	29/11/2012	ERS6404375
kpnneo_47	*Klebsiella pneumoniae*	NEOMERO1	Lithuania	12/02/2014	ERS6404368
eclneo_48	*Enterobacter cloacae*	NEONIN	UK	28/03/2014	ERS6404370
econeo_49	*Escherichia coli*	NEONIN	UK	27/05/2014	ERS6404371
econeo_50	*Escherichia coli*	NEONIN	UK	30/05/2014	ERS6404373
econeo_51	*Escherichia coli*	NEONIN	UK	03/06/2014	ERS6404366
smaneo_52	*Serratia marcescens*	NEONIN	UK	25/05/2014	ERS6404378
eclneo_53	*Enterobacter cloacae*	NEONIN	UK	24/07/2014	ERS6404354
econeo_54	*Escherichia coli*	NEONIN	UK	13/03/2014	ERS6404377
econeo_55	*Escherichia coli*	NEONIN	UK	02/07/2014	ERS6404383
kpnneo_56	*Klebsiella pneumoniae*	NEONIN	UK	04/04/2014	ERS6404381
eclneo_57	*Enterobacter cloacae*	NEONIN	UK	17/08/2012	ERS6404369
econeo_58	*Escherichia coli*	NEONIN	UK	26/11/2012	ERS6404382
ekoneo_59	*Enterobacter kobei*	NEONIN	UK	25/08/2012	ERS6404355
econeo_60	*Escherichia coli*	NEONIN	UK	09/11/2012	ERS6404347
eclneo_61	*Enterobacter cloacae*	NEONIN	UK	18/02/2013	ERS6404386
smaneo_62	*Serratia marcescens*	NEONIN	UK	19/02/2013	ERS6404387
smaneo_63	*Serratia marcescens*	NEONIN	UK	18/03/2013	ERS6404388

koxneo_64	*Klebsiella oxytoca*	NEONIN	UK	08/04/2013	ERS6404390
kpnneo_65	*Klebsiella pneumoniae*	NEONIN	UK	03/09/2013	ERS6404391
econeo_66	*Escherichia coli*	NEONIN	UK	19/11/2013	ERS6404389
econeo_67	*Escherichia coli*	NEONIN	UK	06/11/2013	ERS6404392
econeo_68	*Escherichia coli*	NEONIN	UK	09/02/2010	ERS6404400
econeo_69	*Escherichia coli*	NEONIN	UK	24/03/2010	ERS6404398
econeo_70	*Escherichia coli*	NEONIN	UK	13/06/2010	ERS6404394
econeo_71	*Escherichia coli*	NEONIN	UK	20/06/2010	ERS6404395
smaneo_72	*Serratia marcescens*	NEONIN	UK	04/07/2010	ERS6404396
econeo_73	*Escherichia coli*	NEONIN	UK	12/10/2010	ERS6404397
eclneo_74	*Enterobacter cloacae*	NEONIN	UK	18/10/2010	ERS6404393
smaneo_75	*Serratia marcescens*	NEONIN	UK	08/12/2010	ERS6404401
econeo_76	*Escherichia coli*	NEONIN	UK	12/12/2010	ERS6404399
econeo_77	*Escherichia coli*	NEONIN	UK	27/01/2011	ERS6404402
econeo_78	*Escherichia coli*	NEONIN	UK	02/03/2011	ERS6404403
econeo_79	*Escherichia coli*	NEONIN	UK	06/03/2011	ERS6404404
eclneo_80	*Enterobacter cloacae*	NEONIN	UK	05/08/2011	ERS6404405
smaneo_81	*Serratia marcescens*	NEONIN	UK	25/09/2011	ERS6404406
econeo_82	*Escherichia coli*	NEONIN	UK	10/10/2011	ERS6404407
eclneo_83	*Enterobacter cloacae*	NEONIN	UK	31/12/2011	ERS6404411
econeo_84	*Escherichia coli*	NEONIN	UK	15/02/2012	ERS6404412
econeo_85	*Escherichia coli*	NEONIN	UK	10/06/2012	ERS6404408
econeo_86	*Escherichia coli*	NEONIN	UK	30/06/2013	ERS6404409
econeo_87	*Escherichia coli*	NEONIN	UK	23/08/2012	ERS6404410
